# Healthy Lifestyle in Children and Adolescents and Its Association with Subjective Health Complaints: Findings from 37 Countries and Regions from the HBSC Study

**DOI:** 10.3390/ijerph16183292

**Published:** 2019-09-07

**Authors:** Adilson Marques, Yolanda Demetriou, Riki Tesler, Élvio R. Gouveia, Miguel Peralta, Margarida Gaspar de Matos

**Affiliations:** 1 Centro Interdisciplinar do Estudo da Performance Humana, Faculdade de Motricidade Humana, Universidade de Lisboa, 1499-002 Lisboa, Portugal; 2 Instituto de Saúde Ambiental, Faculdade de Medicina, Universidade de Lisboa, 1649-028 Lisboa, Portugal; 3 Department of Sports and Health Sciences, Technical University of Munich, 80333 Munich, Germany; 4 Department of Health Systems Management, Faculty of Health Sciences, Ariel University, Ariel 40700, Israel,; 5 Department of Physical Education and Sport, University of Madeira, 9000-082 Funchal, Portugal,; 6 Madeira Interactive Technologies Institute, 9020-105 Funchal, Portugal; 7 Faculdade de Motricidade Humana, Universidade de Lisboa, 1499-002 Lisboa, Portugal

**Keywords:** school-aged children, health, behaviors, lifestyle, HBSC

## Abstract

Background: It is important to clearly understand the factors associated with subjective health complaints. The study aimed to investigate the relationship between subjective health complaints, several health behaviors, and a composite measure of healthy lifestyle. Methods: Data were from the Health Behaviour in School-aged Children (HBSC) 2014 international database. Participants were 167,021 children and adolescents, aged 10–16 years, from 37 countries and regions. A composite score of healthy lifestyle was created using a combination of daily physical activity, daily consumption of fruit and vegetables, <2 hours spent daily in screen-based behaviors, no drinking, and no smoking. The subjective health complaints assessed were headaches, stomach aches, backache, dizziness, feeling low, irritability, nervousness, and sleep difficulties. Results: Those who engage in physical activity every day, spend less than two hours a day in screen-based behaviors, do not drink alcohol, and do not smoke tobacco presented a higher likelihood of not having subjective health complaints. A healthy lifestyle was significantly related to having less of all the subjective health complaints. Those with a healthy lifestyle were 50% (OR = 0.5, 95% CI: 0.5–0.6, p < 0.001) less likely to have multiple health complaints. Conclusions: Healthy behaviors and healthy lifestyles are related with less subjective health complaints and less multiple health complaints.

## 1. Introduction

During childhood and adolescence, subjective somatic and psychological complaints such as headaches, stomach aches, backaches, feeling dizzy, feeling low, being irritated, feeling nervous or having difficulties in sleeping are common [1]. Subjective health complaints increase with rising age, mainly among girls and youth from lower socioeconomic status [2,3,4,5].

Subjective health complaints can be defined as symptoms experienced with or without a medical diagnosis, and this represents a shift in the criteria used to evaluate people’s health outcomes. The traditional endpoints used by physicians, such as clinical observation, function test or laboratory values, have also been complemented, or substituted in some cases by people-oriented outcomes [6,7,8]. Reported subjective health complaints constitute a public health concern because subjective health feelings are linked to health outcomes [6,7,8]. Among adolescents, subjective health complaints are also related to dropping-out of school [9] and consequently to lower levels of education in adulthood [10], representing a functional significance. In addition, subjective health complaints are associated with physical wellbeing, are an indicator of mental ill health, and may become chronic pain symptoms and psychological disorders in adulthood [5].

As a public health concern, it is important to have a better understanding of the factors associated with subjective health complaints in order to develop strategies to decrease their prevalence in childhood and adolescence, once there is a connection with their health status. Moreover, in this age group, behaviors are main determinants of health [11]. The most important behaviors associated with children’s and adolescents’ health status are in daily physical activity, spending less than two hours in screen-based sedentary time, having a healthy diet, and abstaining from alcohol and tobacco consumption [12,13]. Literature has shown that engaging in physical activity and spending less time in screen-based sedentary behaviors are related to fewer health complaints [14,15,16]. Similarly, smoking and alcohol consumption are substantially associated with the adolescents’ subjective health and could affect the quality of life and lead to a greater burden of chronic diseases in the future [5,17]. However, thus far, a measure has not been created that expresses a healthy lifestyle and establishes a relationship with adolescents’ subjective health complaints, using a combination of several health behaviors.

According to the self-determination theory (SDT), motivation adopted for different activities (e.g., partaking in physical activity or maintaining a healthy diet) depends on the environmental support of individuals’ three psychological needs: autonomy, competence, and relatedness [18]. Satisfaction from these needs depends on environmental support [19,20,21]. SDT considers that motivation is dependent on context, while emphasizing the role of the environment in motivational change [22]. Therefore, according to SDT, significant individuals (e.g., teachers or parents) take on a primary role in supporting children’s and adolescents’ psychological needs, contributing to their motivation for various activities.

Behaviors supporting autonomy include showing an understanding for another person’s perspective and offering choices. Behaviors supporting competence include setting challenging goals and providing informative feedback. Behaviors supporting relatedness include empathy and acceptance, in addition to minimizing competition [23]. According to SDT, when these behaviors are conducted by parents in the context of their adolescent’s physical activity or dieting behavior, it impacts their children’s motivation to be involved in the physical activity or diet process, and plays a role in the success of partaking in physical activities and diet habits.

Findings from studies have shown that when engaging in activities for autonomous reasons (i.e., enjoying it or view as important), individuals achieve better results and manifest higher wellbeing. When tasks are taken on for less autonomous reasons (e.g., to please others or avoid punishment), they have been shown to produce lower achievement and wellbeing [24].

Joining various health behaviors to determine a healthy lifestyle measure is an asset that may improve public health guidelines. It could also represent a paradigm shift in the way people think about lifestyle as a combination of several healthy behaviors. Therefore, the present study aimed to investigate the relationship between children’s and adolescents’ subjective health complaints, several health behaviors, and a composite measure of healthy lifestyle. For this study, we used a representative wide sample of adolescents from 37 countries and regions in Europe, Israel, and North America (Canada). To the best of our knowledge, this is the first study examining the relationship between subjective health complaints and a healthy lifestyle.

## 2. Materials and Methods

### 2.1. Participants and Procedures

Data was retrieved from the Health Behavior in School-aged Children (HBSC) 2014 international database. The methods and design of the HBSC are described elsewhere [25,26]. Briefly, the HBSC is a World Health Organization collaborative study that is conducted every 4 years in several European and Northern American countries. The HBSC population target are children and adolescents aged 11, 13 and 15, who attend regular schools. The study examines health behaviors and lifestyle of young people, aiming to gain an understanding of their health status and well-being. The survey is conducted in accordance with the ethical guidelines from ethical committees from each country. School administrators in each country give their consent, and legal guardians give written informed consent. Participation is anonymous and the administration of the surveys is conducted according to the HBSC standard survey protocol [25]. The HBSC 2014 survey sample consisted of 214,080 pupils (105,414 boys and 108666 girls) attending grades 6, 8 and 10. For the present study, eligible pupils were those who reported subjective health complaints (n = 198,386) and the following behaviors: physical activity levels (n = 207,980), screen-based sedentary behaviors (n = 188,491), eating fruit and vegetables (n = 207,966), and alcohol (n = 203,383) and tobacco consumption (n = 210,395). The result was a final sample size of 167,021 pupils (80,558 boys and 86,463 girls) who reported both subjective health complaints and all heath behaviors, aged 10–16 (mean = 13.6 ± 1.6), from 37 countries and regions (Albania, Austria, Flemish Belgium, French Belgium, Bulgaria, Canada, Croatia, Czech Republic, Denmark, England, Estonia, France, Germany, Greece, Hungary, Iceland, Ireland, Israel, Italy, Latvia, Luxembourg, Macedonia, Malta, Moldova, the Netherlands, Norway, Poland, Portugal, Romania, Russia, Scotland, Slovakia, Slovenia, Spain, Sweden, Switzerland, and Wales). Ethical approval was sought from the universities, ethics boards, and other authorities associated with the research team in each country.

### 2.2. Measures

#### 2.2.1. Socio-Demographic Characteristics

Pupils reported their gender, age, and school grade. In order to assess socioeconomic status, the family affluence scale was used, which consists of asking pupils about family car ownership, bedroom for themselves, holidays, and the number of computers at home. Afterwards, a composite score was calculated based on responses to these four items, and a three-point ordinal scale was obtained: low (0 to 2), middle (3 to 5), and high (6 to 9). The family affluence scale is a valid measure for adolescents’ wealth, and a proxy of socioeconomic status [27].

#### 2.2.2. Healthy Lifestyle Behaviors and Healthy Lifestyle Composite Score

To assess physical activity, pupils were requested to rate the number of days over the past week during which they were physically active for a total of at least 60 minutes per day. Answers were given on an 8-point scale (0 = none to 7 = daily). Responses were dichotomized into ≥6 times per week and daily, according to the physical activity guidelines [28]. Pupils were asked to indicate the customary time (hours per day) that they spend watching television, playing videogames, and using the computer. Total screen-based behavior was calculated by the sum of these behaviors. The sum of screen-based sedentary behaviors was dichotomized into ≥2 hours and <2 hours daily [29]. Boys and girls were asked to report the frequency with which they eat fruit and vegetables. The options were “never”, “less than once a week”, “once a week”, “2–4 days a week”, “5–6 days a week”, “once every day” and “several times every day”. The two items were dichotomised into daily and less than daily because fruit and vegetable are recommend to be eaten every day [30,31]. Pupils were asked how often they drank alcohol drinks, such as beer, wine, and liquor/spirits. For each alcoholic drink, response options were “never,” “rarely,” “every month,” “every week,” and “every day.” Because alcohol consumption is harmful to adolescents’ health [32], responses were dichotomised into drinking (regardless of frequency) and never drinking. Tobacco consumption was assessed according to question “how often do you smoke tobacco at present?”. Responses options were “every day”, “at least once a week, but not every day”, “less than once a week” or “never”. Because there is no threshold of safety for smoking, responses were recoded into current smoker (regularly or sometimes), and non-smoker.

The healthy lifestyle composite score was created by combining all these healthy behaviors. Pupils scored one point for achieving each of the following healthy lifestyle categories: a) daily physical activity b) daily consumption of fruit and vegetables c) spent <2 daily hours in screen-based sedentary behaviors, d) never drinking, and e) never smoking. Thus, the healthy lifestyle score ranged from 0 to 5, with only a score of 5 representing a healthy lifestyle.

#### 2.2.3. Subjective Health Complaints

The subjective health complaints assessed were headaches, stomach aches, backache, dizziness, feeling low, irritability, nervousness, and sleep difficulties. Children and adolescents were asked to report the frequency of subjective health complaints during the last six months on a five-point scale ranging from 1 = ”rarely or never” to 5 = ”about every day”.

### 2.3. Data Analysis

Subjective health complaints can be divided as somatic complaints (headache, stomach ache, backache, dizziness) and psychological complaints (feeling low, irritability, nervousness, sleep difficulties) [33,34], and used together to measure a one-dimensional latent trait of psychosomatic complaints [35]. An exploratory factor analysis and the Cronbach’s Alpha, performed to investigate the underlying structure for subjective health complaints [33,34,35], showed that the solution reflected somatic health complaints (α = 0.705), psychological health complaints (α = 0.722), and psychosomatic health complaints (α = 0.811). Therefore, three new variables were computed to calculate the symptoms’ scores. Higher values indicated higher subjective health complaints, but the scales were reversed. In order to facilitate the interpretation of these variables, the following transformation metric was applied: t_i_ = (z_i_ × 5) + 10. This maps the z-scores for each variable onto ‘10’ as the mean value and ‘5’ as the standard deviation. Also, a multiple health complaints variable was created, representing those who reported two or more symptoms more than once a week in the past six months [2,36].

Descriptive statistics were calculated (means, standard deviation, and percentages) for all variables. Independent Chi-Square test and ANOVA were used to analyze the relationship between subjective health complaints, healthy behaviors, healthy lifestyle score and the pupils’ age groups. Two models of logistic binary regression were conducted to analyze the effect of the healthy lifestyle score on adolescents’ health complaints, and multiple health complaints. Model 1 was unadjusted, and model 2 was adjusted for family affluence scale and self-rated health. Finally, a linear regression was conducted to analyze the relationship between healthy lifestyle score with somatic symptoms, psychological symptoms, and psychosomatic symptoms. A significant interactions effect was observed for sex, age, and healthy lifestyle. Thus, the analysis was stratified according to sex and age groups. Statistical analysis was performed using SPSS 25 (IBM, Chicago, IL, USA). The significance level was set at *p* < 0.05.

## 3. Results

Table 1 presents the characteristics of the study sample. The prevalence of subjective health complaints ranged from 10.4% (95% CI: 10.3, 10.6) for dizziness to 22.8% (95% CI: 22.6, 23.0) for irritability, and 33.4% (95% CI: 33.2, 33.6) presented multiple health complaints. About 20% (19.6%, 95% CI: 19.4, 19.8) of adolescents engage in physical activity every day, 26.3% (95% CI: 26.1, 26.5) spend less than two hours a day in screen-based behaviors, and 23.4% (95% CI: 23.2, 23.6) had daily fruit and vegetables consumption. On the other hand, 61.1% (95% CI: 60.8, 61.3) do not drink alcohol and 92.1% (95% CI: 92.0, 92.3) do not smoke tobacco. Only 1.9% (95% CI: 1.8, 1.9) of the adolescents presented a healthy lifestyle, by adopting all of these health-related behaviors.

The relationship between subjective health complaints, healthy behaviors, healthy lifestyle and age groups are presented in Table 2. In general, as age increases, there are more health complaints among both boys and girls. Furthermore, for boys, multiple complaints increase significantly from 23.5% (95% CI: 22.4, 24.5) by the age of 10-12 to 27.3% (95% CI: 26.3, 28.4) at the age of 15–16. Among girls, the increase is more pronounced, from 30.8% (95% CI: 29.8, 31.7) by the age of 10–12 to 50.2% (95% CI: 49.3, 51.0) at the age of 15–16. Similarly, there is also a significant increase in the level of somatic, psychological, and psychosomatic symptoms. On the other hand, as age increases, the number of children and adolescents who engage in healthy behaviors decrease. The average of healthy behaviors decrease from 2.6 (95% CI: 2.6, 2.6) to 1.8 (95% CI: 1.7, 1.8) for the boys and from 2.8 (95% CI: 2.8, 2.8) to 1.8 (95% CI: 1.8, 1.8) for the girls.

Table 3 presents the association between the healthy lifestyle score and pupils’ health complaints. For boys and girls, in all age groups, it is observed that a higher healthy lifestyle score decreases the likelihood of children and adolescents having health complaints more than once a week. This was observed in the crude model, and even when the analysis was adjusted for the family affluence scale and self-rated health, the results remained similar.

Results of the relationship between healthy lifestyle score and psychosomatic symptoms are presented in Table 4. A higher healthy lifestyle score is negatively correlated with somatic, psychological, and psychosomatic symptoms, among boys and girls, in each age group. The observed correlation remained significant, even after adjusting the model for family affluence scale and self-rated health.

**Figure 1:**
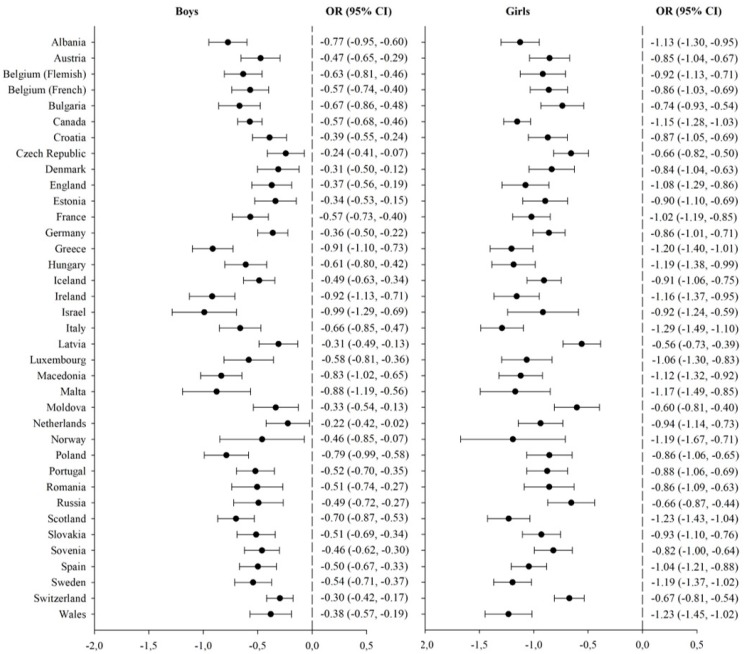
Relationship between psychosomatic symptoms and a healthy lifestyle score, by sex and country.

The relationship between psychosomatic symptoms and a healthy lifestyle score, by sex and country, is shown in Figure 1. Regardless of sex and country, a higher healthy lifestyle score is significantly and negatively correlated with psychosomatic symptoms. Among boys, a higher decrease in symptoms when leading a healthy lifestyle was especially observed in Greece (β = –0.91, 95% CI: –1.10, –0.73), Ireland (β = –0.92, 95% CI: –1.13, –0.71), and Israel (β = –0.99, 95% CI: –1.29, –0.69). Among girls, the healthy lifestyle score had a higher impact on children and adolescents from Scotland (β = –1.23, 95% CI: –1.43, –1.04), Switzerland (β = –1.23, 95% CI: –1.44, –1.02), and Italy (β = –1.29, 95% CI: –1.49, –1.10).

## 4. Discussion

This study results showed that subjective health complaints are frequent during adolescence, with almost one-third reporting multiple health complaints. Irritability is the most common complaint. On the other hand, only 1.9% of the adolescents presented a healthy lifestyle (i.e. those who achieved all five healthy behaviors). This study adds novel information to the literature, showing that both individual healthy behaviors and a healthy lifestyle (a combination of individual health behaviors) behavior were related with fewer subjective health complaints and fewer multiple health complaints.

Both the benefits of physical activity and the harmful effect of screen-based sedentary behaviors on health are well documented [37,38]. Physical activity is related with less health complaints, such as irritability, nervousness, and musculoskeletal complaints [14,39]. On the other hand, there is a positive association between screen-based behaviors, musculoskeletal pain, backache, depression, headache, feeling low, irritability, feeling nervous, and sleep problems [14,40,41,42]. It was thus expected that physical activity could be related to less subjective health complaints, as previously reported [14,15].

The present study corroborates the literature, specifically in the relationship between physical activity, screen-based behaviors, and subjective health complaints. Physical activity and screen-based behaviors are associated with increased wellbeing and subjective health [43], which in turn relate to subjective health complains. Furthermore, regular physical activity is known to improve the overall health status. It also reinforces the importance of promoting physical activity and reducing screen-based behaviors among adolescents [29], in agreement with World Health Organization recommendations [28]. Such suggestions aim to help reduce the number of reports of subjective health complaints (somatic symptoms, psychological symptoms, psychosomatic symptoms) and latent unidimensional psychosomatic symptoms.

Eating fruit and vegetables reduces the risk of obesity, improves physical fitness, and provides protection from a variety of diseases, such as cardiovascular diseases, diabetes, and some types of cancers [44,45,46]. The daily consumption of fruit and vegetables is therefore highly recommended [31]. Although there is wide information on the health benefits of eating fruit and vegetables, so far there are no studies that have analyzed its relationship with subjective health complaints among adolescents.

The results of this study highlight the fact that eating fruit and vegetables every day may help decrease the number of health symptoms, such as headaches, dizziness, feeling low, irritability, and nervousness. Furthermore, eating fruit and vegetables daily is related with less somatic symptoms, psychological symptoms, and general psychosomatic symptoms. In spite of the importance of fruit and vegetables for general human nutrition and the prevention of chronic diseases [44], this study underlines their importance for the reduction of health complaints, which are linked to health outcomes [6,7,8].

An alcohol free and smoke free lifestyle is the healthiest choice for youth [47]. Alcohol consumption is harmful for children’s and adolescents’ health [32]; even drinking less than once a week during adolescence is associated with higher risks of mental health problems [48,49]. Smoking during adolescence is associated with somatic and psychological health problems, such as respiratory problems, headaches, stomach aches, muscular pain, fatigue, nervousness, anxiety, and sleeping problems [17,50]. Furthermore, smokers frequently have more health complaints than non-smokers [51]. The opposite is also suggested by Wong et al. [52], as subjective health complaints reflect a lack of well-being and health, and are an antecedent to problem behaviors such as smoking. The increasing number of health complaints related to alcohol and tobacco consumption calls attention to its harmful effects. It can thus be suggested that the elimination or reduction of alcohol and tobacco consumption may improve quality of life by decreasing pupils’ psychosomatic symptoms.

The consistency of a healthy lifestyle in reducing all health complaints to less than once a week and its relationship with less somatic, psychological, and psychosomatic symptoms showed that the combination of several healthy behaviors could be a composite measure of a healthy lifestyle, which is a robust variable to analyze the status of children’s and adolescents’ health. Although there is no consensus regarding how to define a healthy lifestyle, it is clear that a composite measure that combines healthy behaviors (engaging in physical activity every day, spending less than two hours per day in screen-based sedentary behaviors, consuming fruit and vegetables daily, and abstaining from alcohol and tobacco consumption) reflects a variable that is linked to better health status. This assumption is in line with the present study’s results and with the fact that these healthy behaviors are amongst the most important ones associated with health [11,12,13]. 

The fact that a healthy lifestyle decreases the odds of children and adolescents having multiple health complaints is noteworthy. Multiple health complaints constitute a significant public health concern across childhood and adolescence. They are associated with impaired emotional and behavioral functioning [53], remarkable disturbances [2], and dropping-out from school [9], indicating an underlying vulnerability to behavioral and emotional problems [2,9,53]. This can be explained by the results, which show a high proportion of adolescents reporting subjective health complaints [2,3,4]. 

This study has several limitations that are important to mention. The HBSC data base misses certain values. Pupils who did not report subjective health complaints or healthy behaviors were excluded (more than 40000 adolescents). In addition, data was self-reported and subject to bias. Concerning data collection, the possible answers for fruit and vegetable intake did not allow to determine the number of portions per day, which would be important for a more accurate assessment of consumption according to prescribed guidelines [30]. The HBSC survey did not have any questions assessing sleep duration, and then it was not included as an important variable for calculating pupils’ healthy lifestyles [54]. Furthermore, the cross-sectional study design precludes causal inference for the relationship between healthy lifestyle, subjective health complaints, and multiple health complaints. Lastly, possible differences between young people from the several countries participating in the study were not considered. This was due to the participation of several countries and the aim of the study was to examine young people as an overall group.

In the light of these results, we suggest the need for a more holistic view on health and lifestyles. The focus should not be on a single behavior, but instead on several actions that together express a way of life. In order to improve public health guidelines, it is important to better understand and promote general determinants of wellbeing that may be associated with the adoption of a healthy lifestyle. Recent research has stressed the need to go beyond “knowledge” and “boutique projects”, and to provide capacities, motivation, and opportunities for young people [55]. This is connected to analyzing both the facilitators of behavior adoptions at the personal, social, and institutional level, and the barriers. This could represent a crucial shift in thinking about lifestyle as a combination of several healthy behaviors. Lastly, the promotion of healthy lifestyles to enhance children’s and adolescents’ health, and consequently their quality of life, could be a public health goal for health professionals and teachers.

## 5. Conclusions

This study shows that only 1.9% of adolescents presented the studied five healthy behaviors at the same time. Individual healthy behaviors and a healthy lifestyle are related to less subjective health complaints and less multiple health complaints. The consistency of a healthy lifestyle in reducing somatic, psychological, and psychosomatic symptoms showed that the combination of several healthy behaviors could be a composite measure of healthy lifestyle, which is a robust variable to analyze pupils’ health status and must be highlighted in public health and education policies.

## Figures and Tables

**Table 1:** Participants’ characteristics (n = 167,021 adolescents).

	Total % or M (95% CI)
Sex	
Boys	48.2 (48.0, 48.5)
Girls	51.8 (51.5, 52.0)
Age (years)	13.6 (13.6, 13.6)
Age	
10–12 years	32.3 (32.1, 32.6)
13–14 years	35.3 (35.1, 35.5)
15–16 years	32.4 (32.2, 32.6)
FAS index	14.0 (14.0, 14.0)
Health symptoms (more than once a week)	
Headache	17.6 (17.5, 17.8)
Stomach ache	11.0 (10.8, 11.1)
Backache	13.3 (13.1, 13.4)
Dizziness	10.4 (10.3, 10.6)
Feeling low	17.7 (17.5, 17.9)
Irritability	22.8 (22.6, 23.0)
Nervousness	22.2 (22.0, 22.4)
Difficulties sleeping	21.7 (21.5, 21.9)
Multiple health complaints (more than once a week)	
Less than 1 complaint	66.6 (66.4, 66.8)
≥2 complaints	33.4 (33.2, 33.6)
Physical activity	
≤6 days/week	80.4 (80.2, 80.6)
Daily	19.6 (19.4, 19.8)
Screen-based behaviors	
≥2 hours/day	73.7 (73.5, 73.9)
<2 hours/day	26.3 (26.1, 26.5)
Fruit and vegetables	
Not daily	76.6 (76.4, 76.8)
Daily	23.4 (23.2, 23.6)
Drink alcohol	
Drink	38.9 (38.7, 39.2)
Do not drink	61.1 (60.8, 61.3)
Smoking	
Smoke	7.9 (7.7, 8.0)
Do not smoke	92.1 (92.0, 92.3)
Healthy lifestyle score	
≤4 healthy behaviors	98.1 (98.1, 98.2)
5 healthy behaviors	1.9 (1.8, 1.9)

%, percentage; M, mean; CI, confidence interval; FAS, family affluence scale.

**Table 2:** Relationship between subjective health complaints, healthy behaviors, and healthy lifestyle computed variable and adolescents age groups.

	Boys (n = 805,58)				Girls (n = 864,63)			
	10–12 years (260,52)	13–14 years (285,65)	15–16 years (259,41)	*p*	10–12 years (279,43)	13–14 years (281,55)	15-16 years (864,63)	*p*
Health symptoms ^1, a^								
Headache	11.9 (10.8, 13.0)	12.1 (11.0, 13.2)	12.1 (11.0, 13.3)	0.719	16.5 (15.4, 17.6)	22.5 (21.5, 23.5)	29.5 (28.6, 30.5)	<0.001
Stomach ache	8.3 (7.1, 9.5)	7.3 (6.2, 8.5)	6.5 (5.3, 7.7)	<0.001	13.3 (12.2, 14.3)	14.1 (13.0, 15.1)	15.5 (14.4, 16.6)	<0.001
Backache	8.5 (7.3, 9.7)	10.8 (9.7, 11.9)	12.9 (11.7, 14.0)	<0.001	10.6 (9.5, 11.7)	15.7 (14.7, 16.7)	20.7 (20.0, 22.1)	<0.001
Dizziness	7.2 (6.0, 8.4)	7.7 (6.6, 8.8)	8.2 (7.0, 9.3)	<0.001	9.2 (8.0, 10.3)	13.7 (12.6, 14.7)	16.2 (15.1, 17.3)	<0.001
Feeling low	10.9 (9.8, 12.1)	11.0 (9.9, 12.1)	13.4 (12.3, 14.5)	<0.001	15.2 (8.0, 10.3)	24.1 (12.6, 14.7)	30.1 (15.1, 17.3)	<0.001
Irritability	15.6 (–1.2, 1.2)	17.8 (16.8, 18.9)	19.8 (18.7, 20.9)	<0.001	18.6 (17.6, 19.7)	28.6 (27.7, 29.6)	35.2 (34.3, 36.2)	<0.001
Nervousness	15.2 (14.1, 16.3)	17.0 (15.9, 18.0)	18.9 (17.8, 20.0)	<0.001	18.3 (17.3, 19.4)	28.3 (27.4, 29.3)	34.2 (33.2, 35.1)	<0.001
Difficulties sleeping	18.4 (17.3, 19.5)	17.6 (16.6, 18.7)	18.2 (17.1, 19.3)	0.056	21.0 (19.9, 22.0)	25.5 (24.6, 26.5)	28.6 (27.6, 29.6)	<0.001
Multiple complaints ^1, a^				<0.001				<0.001
Less than 1 complaint	76.5 (75.9, 77.1)	75.0 (74.4, 75.6)	72.7 (72.0, 73.3)		69.2 (68.6, 69.9)	58.0 (57.3, 58.8)	49.8 (49.0, 50.7)	
≥2 complaints	23.5 (22.4, 24.5)	25.0 (24.0, 26.0)	27.3 (26.3, 28.4)		30.8 (29.8, 31.7)	42.0 (41.1, 42.8)	50.2 (49.3, 51.0)	
Somatic symptoms ^b^	8.7 (8.6, 8.7)	9.1 (9.0, 9.1)	9.4 (9.3, 9.4)	<0.001	9.5 (9.5, 9.6)	10.9 (10.9, 11.0)	12.1 (12.1, 12.2)	<0.001
Psychological symptoms ^b^	8.7 (8.6, 8.8)	9.1 (9.0, 9.1)	9.4 (9.3, 9.4)	<0.001	9.3 (9.3, 9.4)	11.1 (11.0, 11.1)	12.3 (12.2, 13.3)	<0.001
Psychosomatic symptoms ^b^	8.5 (8.5, 8.6)	9.0 (8.9, 9.0)	9.4 (9.4, 9.5)	<0.001	9.3 (9.3, 9.4)	11.1 (11.1, 11.2)	12.4 (12.4, 12.5)	<0.001
Physical activity (daily) ^b^	29.2 (28.1, 30.2)	24.0 (23.0, 25.0)	20.4 (19.3, 21.4)	<0.001	20.0 (18.9, 21.0)	14.6 (13.6, 15.7)	10.6 (9.5, 11.7)	<0.001
Screen-based behaviors (≤2 hours/day)^a^	33.0 (32.0, 34.0)	20.3 (19.3, 21.3)	15.9 (14.8, 17.0)	<0.001	44.2 (43.3, 45.0)	24.9 (23.9, 25.8)	19.4 (18.3, 20.4)	<0.001
Fruit and vegetables (daily) ^a^	23.9 (22.8, 25.0)	19.6 (18.6, 20.7)	18.3 (17.2, 19.4)	<0.001	29.1 (28.1, 30.1)	24.8 (23.8, 25.8)	24.5 (23.5, 25.5)	<0.001
Drink alcohol (do not drink) ^a^	74.5 (73.8, 75.1)	60.5 (59.8, 61.3)	37.2 (36.2, 38.1)	<0.001	84.9 (84.4, 85.3)	67.2 (66.5, 67.8)	41.0 (40.1, 41.9)	<0.001
Smoking (do not smoke) ^a^	98.0 (97.9, 98.2)	94.2 (93.9, 94.5)	84.0 (83.5, 84.5)	<0.001	98.9 (98.8, 99.0)	94.0 (93.7, 94.3)	83.5 (83.0, 83.9)	<0.001
Healthy lifestyle (5 healthy behaviors) ^a^	3.2 (2.0, 4.4)	1.3 (0.2, 2.5)	0.7 (–0.5, 2.0)	<0.001	3.8 (2.6, 4.9)	1.5 (0.4, 2.6)	0.7 (–0.5, 1.8)	<0.001
Health lifestyle score ^b^	2.6 (2.6, 2.6)	2.2 (2.2, 2.2)	1.8 (1.7, 1.8)	<0.001	2.8 (2.8, 2.8)	2.3 (2.2, 2.3)	1.8 (1.8, 1.8)	<0.001

Abbreviation: M, mean; SD, standard deviation; FAS, family affluence scale. ^1^ Health symptoms and multiple health complaints were more than once a week. Differences were tested by Qui-square and ANOVA. ^a^ % (95% CI). ^b^ Mean (95% CI).

**Table 3:** Association between healthy lifestyle score and adolescents’ health complaints.

Health Complaints More than Once a Week	Healthy Lifestyle Score OR (95% CI)					
	Model 1			Model 2		
Boys	10–12 years	13–14 years	15–16 years	10–12 years	13–14 years	15–16 years
Headache	0.82 (0.78, 0.85)	0.81 (0.78, 0.84)	0.82 (0.79, 0.85)	0.88 (0.84, 0.92)	0.87 (0.84, 0.91)	0.88 (0.84, 0.92)
Stomach ache	0.80 (0.77, 0.84)	0.81 (0.77, 0.85)	0.81 (0.77, 0.86)	0.87 (0.83, 0.91)	0.88 (0.83, 0.92)	0.88 (0.83, 0.93)
Backache	0.81 (0.77, 0.85)	0.83 (0.80, 0.86)	0.86 (0.82, 0.89)	0.87 (0.83, 0.91)	0.88 (0.84, 0.92)	0.91 (0.88, 0.95)
Dizziness	0.79 (0.75, 0.83)	0.83 (0.79, 0.87)	0.82 (0.78, 0.86)	0.86 (0.81, 0.91)	0.90 (0.86, 0.94)	0.89 (0.85, 0.93)
Feeling low	0.79 (0.76, 0.82)	0.77 (0.74, 0.81)	0.81 (0.78, 0.85)	0.86 (0.83, 0.90)	0.84 (0.81, 0.88)	0.89 (0.86, 0.93)
Irritability	0.75 (0.73, 0.78)	0.75 (0.73, 0.78)	0.79 (0.77, 0.82)	0.81 (0.78, 0.84)	0.80 (0.78, 0.83)	0.85 (0.82, 0.88)
Nervousness	0.79 (0.77, 0.82)	0.80 (0.78, 0.83)	0.81 (0.78, 0.83)	0.84 (0.81, 0.87)	0.85 (0.82, 0.88)	0.85 (0.83, 0.88)
Difficulties sleeping	0.83 (0.80, 0.85)	0.84 (0.81, 0.86)	0.86 (0.83, 0.88)	0.88 (0.85, 0.91)	0.89 (0.86, 0.92)	0.92 (0.89, 0.95)
Multiple health complaints	0.76 (0.73, 0.78)	0.78 (0.75, 0.80)	0.79 (0.77, 0.81)	0.82 (0.79, 0.85)	0.84 (0.81, 0.86)	0.85 (0.83, 0.88)
Girls	10–12 years	13–14 years	15–16 years	10–12 years	13–14 years	15–16 years
Headache	0.82 (0.80, 0.85)	0.75 (0.73, 0.78)	0.80 (0.78, 0.82)	0.90 (0.87, 0.94)	0.83 (0.81, 0.86)	0.86 (0.84, 0.89)
Stomach ache	0.85 (0.82, 0.88)	0.74 (0.72, 0.77)	0.76 (0.74, 0.79)	0.88 (0.88, 0.96)	0.81 (0.79, 0.84)	0.83 (0.80, 0.86)
Backache	0.83 (0.80, 0.87)	0.80 (0.77, 0.83)	0.85 (0.83, 0.88)	0.91 (0.87, 0.95)	0.87 (0.84, 0.89)	0.92 (0.89, 0.95)
Dizziness	0.82 (0.78, 0.86)	0.74 (0.71, 0.76)	0.82 (0.79, 0.84)	0.92 (0.87, 0.96)	0.82 (0.79, 0.85)	0.90 (0.87, 0.93)
Feeling low	0.77 (0.74, 0.79)	0.66 (0.64, 0.68)	0.74 (0.72, 0.76)	0.85 (0.82, 0.87)	0.73 (0.71, 0.76)	0.81 (0.79, 0.83)
Irritability	0.75 (0.72, 0.77)	0.66 (0.64, 0.68)	0.73 (0.71, 0.74)	0.81 (0.78, 0.84)	0.73 (0.69, 0.73)	0.78 (0.76, 0.80)
Nervousness	0.78 (0.75, 0.80)	0.73 (0.71, 0.75)	0.80 (0.78, 0.82)	0.83 (0.80, 0.86)	0.78 (0.76, 0.81)	0.86 (0.84, 0.88)
Difficulties sleeping	0.84 (0.81, 0.86)	0.77 (0.75, 0.79)	0.82 (0.79, 0.84)	0.90 (0.87, 0.93)	0.83 (0.80, 0.85)	0.87 (0.85, 0.86)
Multiple health complaints	0.76 (0.74, 0.78)	0.68 (0.66, 0.70)	0.75 (0.73, 0.76)	0.84 (0.81, 0.86)	0.74 (0.72, 0.76)	0.81 (0.79, 0.83)

Abbreviation: OR, odds ratio; CI, confidence interval. Less than once a week is the reference category for all adolescents’ health complaints and multiple health complaints. Model 1: unadjusted. Model 2: analysis were adjusted for family affluence scale and self-rated health.

**Table 4:** Relationship between healthy lifestyle score and psychosomatic symptoms.

	Healthy Lifestyle Score β (95% CI)					
	Model 1			Model 2		
Boys	10–12 years	13–14 years	15–16 years	10–12 years	13–14 years	15–16 years
Somatic symptoms	–0.54 (–0.60, –0.49)	–0.94 (–1.00, –0.88)	–0.81 (–0.87, –0.75)	–0.37 (–0.43, –0.32)	–0.38 (–0.43, –0.32)	–0.33 (–0.38, –0.27)
Psychological symptoms	–0.64 (–0.70, –0.58)	–0.69 (–0.75, –0.64)	–0.66 (–0.71, –0.60)	–0.46 (–0.52. –0.40)	–0.51 (–0.56, –0.46)	–0.45 (–0.51, –0.40)
Psychosomatic symptoms	–0.66 (–0.72, –0.61)	–0.70 (–0.75, –0.65)	–0.66 (–0.72, –0.61)	–0.47 (–0.52, –0.41)	–0.50 (–0.55, –0.45)	–0.44 (–0.49, –0.39)
Girls	10–12 years	13–14 years	15–16 years	10–12 years	13–14 years	15–16 years
Somatic symptoms	–0.60 (–0.66, –0.54)	–0.54 (–0.61, –0.50)	–0.52 (–0.57, –0.46)	–0.35 (–0.41, –0.29)	–0.64 (–0.70, –0.58)	–0.50 (–0.56, –0.45)
Psychological symptoms	–0.79 (–0.85, –0.73)	–1.19 (–1.25, –1.23)	–0.94 (–0.99, –0.88)	–0.55 (–0.61, –0.49)	–0.89 (–0.95, –0.83)	–0.66 (–0.72, –0.60)
Psychosomatic symptoms	–0.78 (–0.84, –0.72)	–1.20 (–1.25, –1.39)	–0.97 (–1.03, –0.92)	–0.51 (–0.57, –0.45)	–0.86 (–0.92, –0.81)	–0.66 (–0.71, –0.60)

Abbreviation: CI, confidence interval. Model 1: unadjusted. Model 2: analysis was adjusted for family affluence scale and self-rated health.
